# The Antimicrobial Activity of Gramicidin A Is Associated with Hydroxyl Radical Formation

**DOI:** 10.1371/journal.pone.0117065

**Published:** 2015-01-26

**Authors:** Je-Wen Liou, Yu-Jiun Hung, Chin-Hao Yang, Yi-Cheng Chen

**Affiliations:** 1 Institute of Biochemistry, Tzu Chi University, Hualien 970, Taiwan; 2 Institute of Medical Sciences, Tzu Chi University, Hualien 970, Taiwan; 3 Department of Medicine, MacKay Medical College, New Taipei City 252, Taiwan; Faculdade de Medicina da Universidade de Lisboa, PORTUGAL

## Abstract

Gramicidin A is an antimicrobial peptide that destroys gram-positive bacteria. The bactericidal mechanism of antimicrobial peptides has been linked to membrane permeation and metabolism disruption as well as interruption of DNA and protein functions. However, the exact bacterial killing mechanism of gramicidin A is not clearly understood. In the present study, we examined the antimicrobial activity of gramicidin A on *Staphylococcus aureus* using biochemical and biophysical methods, including hydroxyl radical and NAD^+^/NADH cycling assays, atomic force microscopy, and Fourier transform infrared spectroscopy. Gramicidin A induced membrane permeabilization and changed the composition of the membrane. The morphology of *Staphylococcus aureus* during gramicidin A destruction was divided into four stages: pore formation, water permeability, bacterial flattening, and lysis. Changes in membrane composition included the destruction of membrane lipids, proteins, and carbohydrates. Most interestingly, we demonstrated that gramicidin A not only caused membrane permeabilization but also induced the formation of hydroxyl radicals, which are a possible end product of the transient depletion of NADH from the tricarboxylic acid cycle. The latter may be the main cause of complete *Staphylococcus aureus* killing. This new finding may provide insight into the underlying bactericidal mechanism of gA.

## Introduction

The driving force for the development of new anti-bacterial drugs is always the inevitable emergence of bacterial resistance to antibiotics following widespread clinical use [1]. The search for new antibiotic drugs has prompted an interest in a group of antimicrobial peptides (AMPs) [2, 3]. The source of AMPs is natural organisms, including animals, plants, or the pathogen itself [3–8]. As part of the innate defense system, AMPs provide protection against a wide variety of microorganisms in both vertebrates and invertebrates [2, 3, 5–7]. Unlike common antibiotic drugs, which in most cases are synthesized by special metabolic pathways, the amino acid sequences of AMPs are naturally encoded in the genetic material of the host organism [3, 5, 6].

The bacterial killing mechanism of AMPs varies and includes membrane permeabilization, metabolism disruption, as well as interruption of DNA and protein functions [3, 9–16]. The most common mechanism of antibacterial activity for linear AMPs, which have mostly an α-helical structure, is membrane permeabilization [2, 17, 18]. Membrane permeabilization by α-helical AMPs has been proposed to proceed via one of two mechanisms: (I) the “barrel-stave” mechanism in which AMPs form pores on the membrane surface [11]; and (II) either the “toroidal pore” or “carpet-like” mechanism in which AMPs act as detergent-like micelles and destroy the membrane [9, 10]. Recently, a common bactericidal mechanism associated with the formation of hydroxyl radicals was proposed [19]. Several studies also demonstrated that, in addition to membrane permeabilization, the bactericidal mechanism of a few AMPs such as polymyxins and pleurocidin may also be associated with the formation of hydroxyl radicals [20, 21].

Gramicidin is an antibiotic peptide synthesized by *Bacillus brevis* that destroys gram-positive bacteria [22, 23]. Unlike most AMPs, gramicidin forms a single ion channel instead of a pore in the membrane [23–28]. The sequence of gramicidin consists of alternating L- and D-amino acids, with the N-terminus modified with a formyl group (-HCO) and the C-terminus conjugated to an aminoethanol group (-NHCH_2_CH_2_OH) [23, 24]. The natural mixture of gramicidin, often denoted as gramicidin D (gD), consists of 80% gramicidin A (gA), 5% gramicidin B (gB), and 15% gramicidin C (gC) [24]. gA has four tryptophan residues at positions 9, 11, 13, and 15. gB and gC differ from gA in the nature of the aromatic residue at position 11, where Trp is replaced by Phe in gB and by Tyr in gC [24].

gA can adopt two major types of folding motifs: the double helix and the helical dimer [25–28]. The latter is referred to as the “channel” form in which gA forms a single channel in model membranes. The size of the gA channel (diameter ∼4 Å) is large enough to accommodate the passage of monovalent cations such as alkaline cations and protons [24–28]. The length of the dimer is ∼26 Å and is within the same order of magnitude as the hydrophobic part of the lipid bilayer [26, 27, 29]. The gA channel has open and closed forms. The open channel of gA is formed by joining two gramicidin monomers to form an N-terminal-to-N-terminal dimer in the middle of the membrane [26, 27]. This open form of the gA channel permits the translocation of ions across the membrane. The closed form of the gA channel is formed when the hydrogen bonds joining the two monomers are broken and the two monomers separate in the bilayer leaflets [26, 27].

The bacterial killing mechanism of gA has a proposed link with the formation of a single gA channel [26, 27, 30]. This is very different from other linear, helical, and pore-forming AMPs such as alamethicin, cecropin, and LL-37 in which the pore is formed by association of a bundle of aggregated peptides [9, 31, 32]. Moreover, a recent study of bacterial killing activity and membrane permeabilization by several AMPs showed that gD has the lowest permeabilization rate (60–70%) but the highest bacterial killing rate (100%) compared to polymyxin B, human neutrophil peptide 1 (hNP-1), and thrombin-induced platelet microbicidal protein 1 (tPMP-1) [30]. Several early studies reported that gramicidin causes mitochondria to become permeable to protons and disrupts respiration [33, 34]. Taken together, evidence suggests that the antimicrobial activity of gA may be different from other AMPs, and membrane permeabilization may not be the sole mechanism involved in gA-induced bacterial killing.

In the present study, we re-examined the bacterial killing mechanism of gA and the antimicrobial effect of gA on the nanostructure and composition changes in the *Staphylococcus aureus* membrane. We demonstrated that the bacterial killing mechanism of gA may involve synergy between hydroxyl radical formation and membrane permeabilization. In particular, the formation of hydroxyl radicals may play an important role in the killing of *S*. *aureus*. Changes in the nanostructure and composition of the *S*. *aureus* cell membrane during destruction were investigated using atomic force microscopy (AFM) and Fourier transform infrared (FT-IR) spectroscopy. Several interesting features were observed for the first time. Our present study may provide insight into the underlying mechanism of the antimicrobial activity of gA.

## Materials and Methods

### Bacterial growth conditions

*S*. *aureus* was grown in 25 ml LB medium in a 250-ml flask at 37°C overnight. Then, an aliquot of the bacterial medium was transferred to 100 ml LB medium to reach an OD_600 nm_ = 0.1. This bacterial medium was then grown to a designated growth phase (OD_600 nm_ = 0.2 for lag phase, OD_600 nm_ = 0.6 for exponential phase, OD_600 nm_ = 1.5 for late-exponential-to-stationary phase). Once the designated growth phase was reached, 25 ml bacterial medium was treated with gA (CALBIOCHEM) at the designated concentration at 37°C. The growth curve of *S*. *aureus* in the presence and absence of gA was measured using an Absorbance Reader MRX II (DYNEX) every 30 min at an optical density of 600 nm.

### FT-IR spectroscopy

For FT-IR spectroscopy, *S*. *aureus* was grown to exponential phase and then treated with the designated concentration of gA. Each sample was washed twice using normal saline (0.85% NaCl) and then coated onto a ZnSe crystal and dried overnight in a laminar flow. The attenuated total reflection (ATR)-FT-IR spectrum was measured using a Perkin Elmer spectrophotometer (PS50) equipped with an ATR compartment. All FT-IR spectra were obtained at wavelengths ranging from 4000 to 800 cm^−1^ with 1024 scans and a resolution of 1 cm^−1^. For every FT-IR spectrum reported, at least three individual samples and three repeated measurements of each sample were taken and averaged.

### Atomic force microscopy (AFM)

All images were collected using an AFM (Nanowizard JPK) that was installed on an inverted optical microscope (Nikon). AFM probes were single-crystal silicon micro cantilevers with 0.02 N/m spring constant (OMCL-TR400PB-1, Olympus, Japan). Samples (10 μl) treated with different concentrations of gA were spotted on freshly cleaved mica (Ted-Pella, Inc.), rinsed with de-ionized water, and dried at room temperature for 24 hr. Images were acquired at scanning rates of 1 to 2 Hz. Analysis of AFM images was performed using JPK and SPIP software.

### Hydroxyl radical assay

All data were collected using a flow cytometer (FACS Calibur, BD) with a 488-nm argon laser and a 515- to 545-nm emission filter (FL1). At least 60,000 bacteria were collected for each sample. To detect hydroxyl radical formation, the fluorescent reporter dye 3′-(*p*-hydroxyphenyl) fluorescein (5 mM) (Invitrogen) was used. In all experiments, samples were taken immediately before addition of different concentrations of gA (time zero) and then every hour for 3 hr. At each time point, approximately 10^6^ cells were collected, washed once, and resuspended in filtered PBS (pH 7.2) prior to measurement.

### NAD^+^/NADH isolation and NAD cycling assay

Either 5 or 10 μg/ml gA was added to *S*. *aureus* culture medium at OD = 0.6 (25 ml in a 250-ml flask), and then 1 ml bacterial sample was taken every 30 min from 0 to 3 hr. These samples were centrifuged at 15,000 ×*g* for 1 min, and the supernatant was discarded. The pellets were collected and immediately frozen in a dry ice-ethanol bath. Seventy-five microliters of 0.2 M HCl (for NAD^+^ extraction) or 0.2 M NaOH (for NADH extraction) was added to the frozen pellets. The samples were then heated in a 100°C sand bath for 10 min and centrifuged at 5,000 ×*g* for 5 min to remove cellular debris. The dinucleotide-containing supernatants were transferred to fresh tubes and stored in the dark on ice until use.

The reaction mixture containing 30 μl of 1.0 M Bicine (pH 8.0), 75 μl sample extract, 75 μl neutralizing buffer (0.1 M HCl for NADH or 0.1 M NaOH for NAD^+^), 30 μl Phenazine Ethosulfate, 30 μl 3-[4,5–dimethylthiazol-2-yl]-2, 5-diphenyltetrazolium bromide (MTT), 30 μl 100% ethanol (Fisher), and 30 μl 40 mM EDTA (pH 8.0) was placed in a 96-well plate and incubated at 30°C for 3 min. Then, 6 μl fresh yeast alcohol dehydrogenase at 500 U/ml in 0.1 M Bicine (pH 8.0) was added to each well to begin the assay. For the NAD^+^ cycling assay, the reduction of MTT was monitored at 570 nm on a microplate reader (FlexStation 3, MD). The reduction rate of MTT was recorded for 10 min at 30°C. The rate of reduction of MTT is proportional to the concentration of dinucleotides in the sample. Dinucleotide standards from 0.05 to 0.75 nM per well were used to calibrate the assay.

## Results

### Effect of gA on bacterial growth

**Fig 1:**
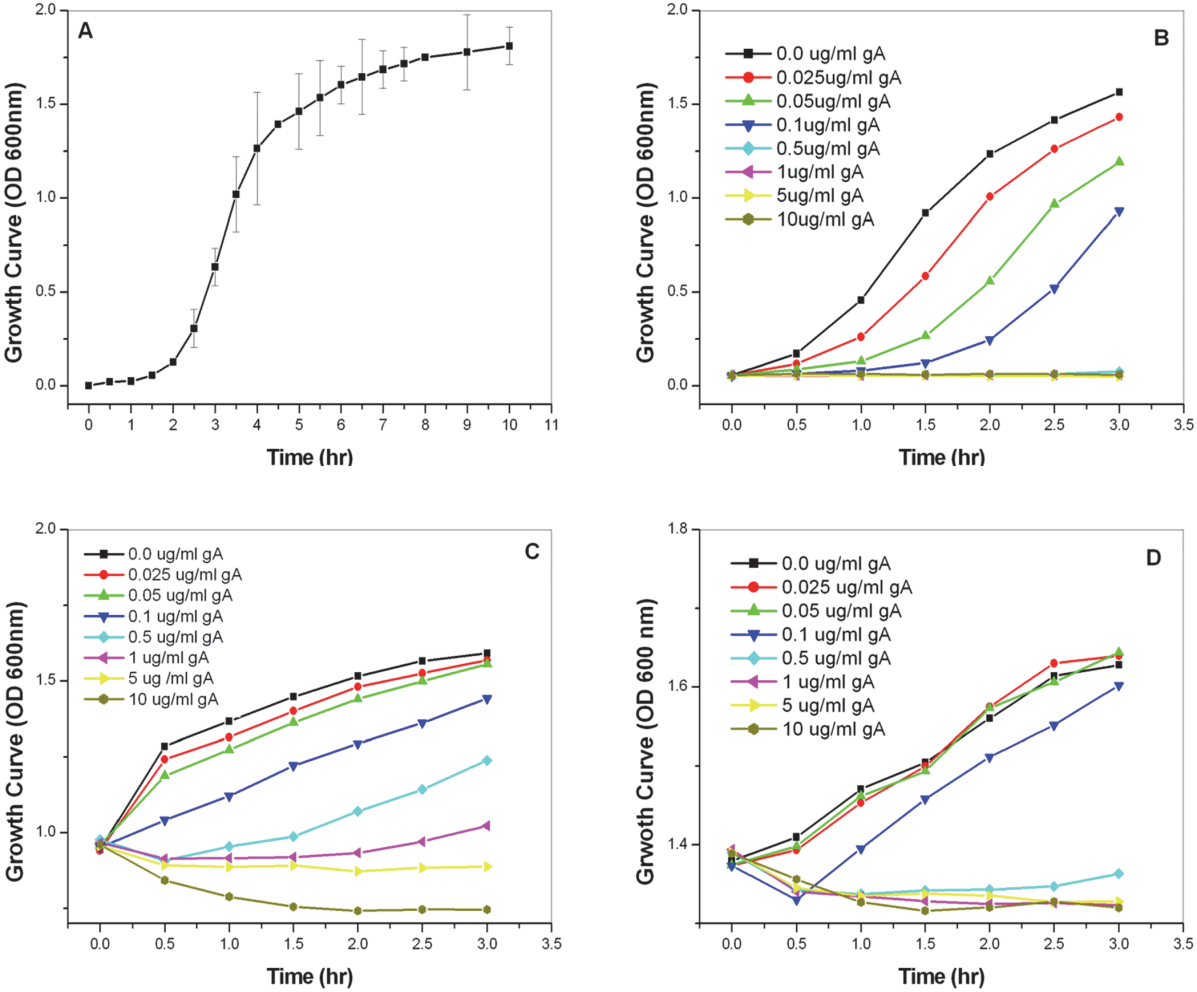
Growth curves of *S. aureus* following treatment with gA. (A) The growth curve of *S. aureus* (A) without treatment with gA. (B-D) The growth curves of *S*. *aureus* in the lag (B), exponential (C), and stationary (D) phases following treatment with different concentrations of gA (0.025–10 μg/ml).

We first examined the effect of gA on the growth of *S*. *aureus*. Fig. 1 (A) shows the growth curve of *S*. *aureus* without gA treatment. The growth of *S*. *aureus* was divided into three phases: lag, exponential, and late-exponential-to-stationary phases. Next, we examined the effect of gA on the growth of *S*. *aureus* by treating bacteria with gA during the three phases. Fig. 1 (B–D) shows the growth curves for *S*. *aureus* treated with different concentrations of gA during the lag, exponential, and stationary phases, respectively. During the lag phase, the growth of *S*. *aureus* was inhibited by gA as shown in Fig. 1 (B) in a dose-dependent manner. The growth of *S*. *aureus* was clearly impeded with increasing gA concentrations. At gA concentrations ≥0.5 μg/ml, the growth of *S*. *aureus* was completely inhibited.

Fig. 1 (C) shows the growth curves for *S*. *aureus* during the exponential phase with or without gA treatment. Bacterial growth was not significantly affected by gA at concentrations ≤0.05 μg/ml. At peptide concentrations ≥0.1 μg/ml, the growth of *S*. *aureus* was significantly inhibited. Fig. 1 (D) shows the growth curve of *S*. *aureus* when treated with gA during the late-exponential-to-stationary phase. When the gA concentration was lower than 0.05 μg/ml, bacterial growth was nearly unaffected by gA, whereas at concentrations ≥0.1 μg/ml, the growth of bacteria was completely inhibited. Taken together, our results demonstrated that the growth of *S*. *aureus* can be efficiently inhibited by gA during all bacterial growth phases.

### Hydroxyl radical generation during treatment with gA

Although the mechanism of bacterial killing by gA has been proposed to be associated with membrane permeabilization, a previous study showed that in a comparison of bacterial killing activity by gD, polymyxin B, hNP-1, and tPMP-1, gD has the lowest permeabilization rate (60–70%) but the highest bacterial killing rate (100%) [30]. This implies that destruction of the membrane may not be the sole mechanism responsible for the bacterial killing ability of gA. Several studies recently indicated that hydroxyl radicals are induced by some chemical bactericidal drugs and other AMPs [19–21]. Therefore, we speculated that gA may also produce hydroxyl radicals. To test this possibility, we measured the formation of hydroxyl radicals during treatment with gA.

**Fig 2:**
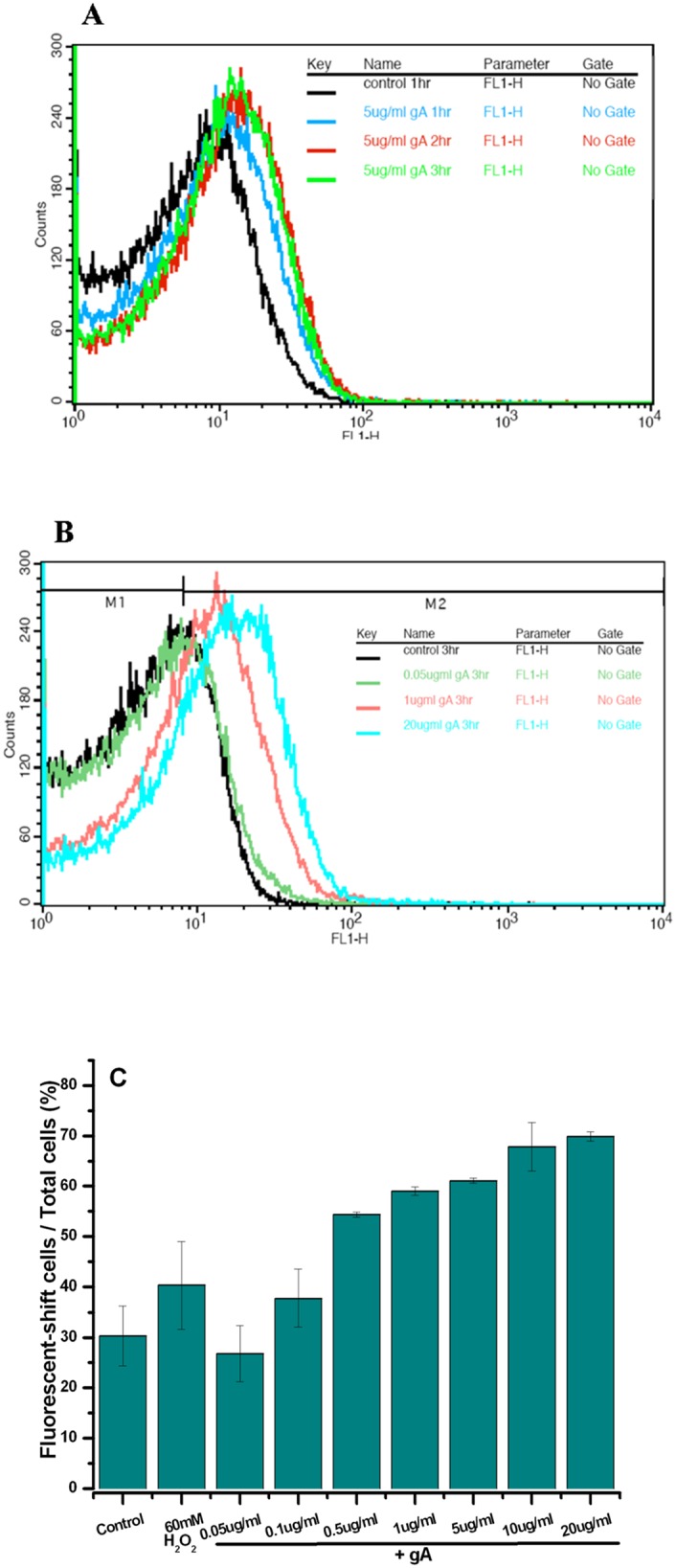
Hydroxyl radical formation following treatment with gA. (A) The hydroxyl radical formation following treatment with 5 μg/ml gA for 1, 2, and 3 hr. (B) The hydroxyl radical formation following treatment with 0, 1, and 20 μg/ml gA for 3 hr. (C) A histogram of hydroxyl radical formation produced by treatment with different concentrations of gA. The hydroxyl radical level following treatment with 60 mM H_2_O_2_ was used as a positive control for comparison.

Fig. 2 (A) shows hydroxyl radical formation during treatment with 5 μg/ml gA for various periods of time. gA clearly induced the production of hydroxyl radicals. The level of hydroxyl radicals increased as time increased and reached a steady-state after treatment with gA for 2 hr. Fig. 2 (B) shows the production of hydroxyl radicals after treatment with different concentrations of gA. At a concentration of 0.05 μg/ml gA, hydroxyl radicals were not induced compared to the control. With a gA concentration higher than 0.5 μg/ml, the production of hydroxyl radicals was significantly induced in a concentration-dependent manner (Fig. 2 (C)). Furthermore, as shown in S1 Fig., the survival rate of *S*. *aureus* with treatment of 0.1 μg gA was rescued when 150 mM of thiourea, a hydroxyl radical scavenger, were added. Taken together, results clearly indicate that the formation of hydroxyl radicals is directly induced by treatment with gA. This result is also consistent with the inhibitory effect of gA on *S*. *aureus* growth. The effective concentration of gA for both inhibition of bacterial growth and formation of hydroxyl radicals was ≥ 0.5 μg/ml. This further indicates that the formation of hydroxyl radicals induced by gA may be another possible cause of bacterial death.

### Mechanism of hydroxyl radical formation

**Fig 3:**
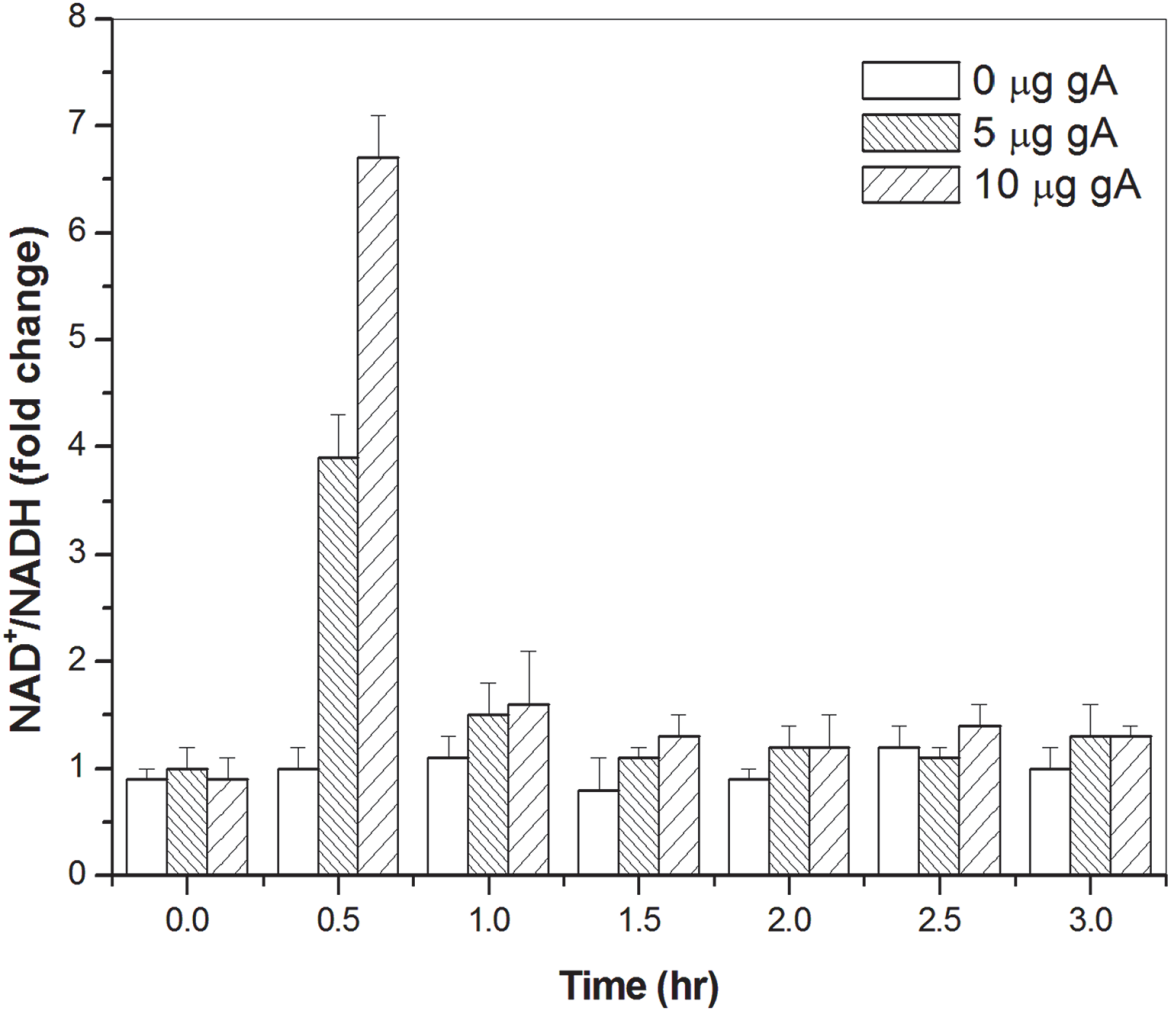
NAD^+^/NADH cycling assay. The NAD^+^/NADH ratio with or without gA treatment. The concentrations of gA used for the assay were 5 and 10 μg/ml.

Previously, several studies have reported that gramicidin disrupts respiration by causing mitochondrial membranes to become permeable to protons [33, 34]. Several bacterial killing activity studies of antibiotics and AMPs also demonstrated that the production of hydroxyl radicals is induced by the Fenton reaction through the reduction of Fe^3+^, the bacterial tricarboxylic acid cycle (TCA) cycle, and the electron transport chain [19–21]. Therefore, we asked if the hydroxyl radicals induced by gA during bacterial killing are also produced via the TCA cycle and a transient depletion of NADH. To test this hypothesis, we performed an NAD^+^ cycling assay to monitor the NAD^+^/NADH level with and without gA treatment (Fig. 3). The ratio of NAD^+^/NADH after gA addition showed a sharp increase at 0.5 hr. A similar significant increase in the NAD^+^/NADH ratio was not observed in the absence of gA. The ratio of NAD^+^/NADH was increased 3.5-fold and 6.7-fold with gA concentrations of 5 μg/ml and 10 μg/ml, respectively. The ratio returned to the untreated level by 1–1.5 hr. Our results are consistent with previous studies [19–21], indicating that the hydroxyl radical formation induced by gA is also associated with disruption of the TCA cycle and a transient depletion of NADH.

### AFM images of *S*. *aureus* treated with gA

Results shown in the previous sections demonstrate that the antimicrobial activity of gA may involve synergy between hydroxyl radical formation and membrane permeabilization. Observations of morphological changes in *S*. *aureus* in the presence of hydroxyl radicals and membrane permeabilization have not been reported. Therefore, AFM was used to observe the morphology of *S*. *aureus* when exposed to hydroxyl radicals.

**Fig 4:**
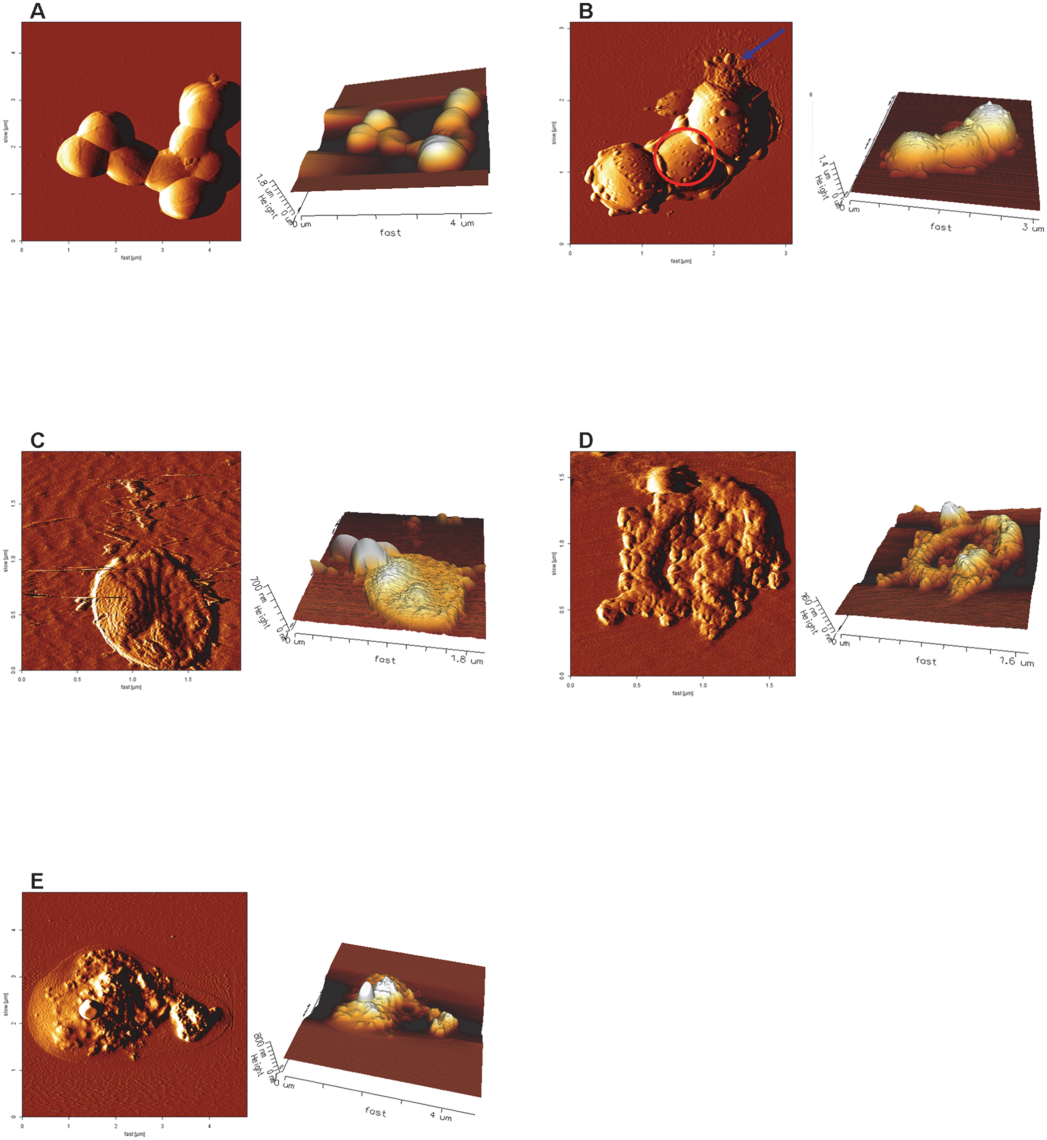
Atomic force microscopic images of *S. aureus* in the exponential phase following treatment with gA. (A) AFM images in the absence of gA treatment, showing typical round *S*. *aureus* cells with a smooth surface. (B-E) AFM images in the presence of treatment with 5 μg/ml gA. In (B), characteristic pores (red circle) and blebs (blue arrow) on the membrane surface of *S*. *aureus* were observed. In (C), flat-shaped *S*. *aureus* cells were observed, indicating that the bacterial membrane was destroyed by treatment with gA. (D) The bacterial membrane was further disrupted. In (E), bacteria were completely destroyed and lysed by treatment with gA.

Several types of morphology for *S*. *aureus* were observed in the presence of gA. Fig. 4 (A) shows the morphology of *S*. *aureus* during the exponential phase without gA treatment. Typical round and aggregated *S*. *aureus* cells were observed. The integrity of the surface of the bacterial membrane was seen without other structures. Following treatment with 5 μg/ml gA, the morphology of *S*. *aureus* was very different from the morphology observed without gA treatment. Fig. 4 (B–E) depicts the four types of damaged *S*. *aureus* morphology. The first type of morphology is depicted in Fig. 4 (B) and consists of characteristic large pores on the membrane surface (red circle). The size of pores varied from 30 to 94 nm, with an average size of 55.6 ± 2.0 nm. In addition to the formation of pores, some parts of the bacterial membrane surface were destroyed, and a few blebs (blue arrow) were also observed.

The second type of morphology is depicted in Fig. 4 (C). An imbalance in permeability was observed. The bacterial membrane was apparently destroyed, and subsequently, bacteria became flattened. Fig. 4 (D) shows the third type of damaged *S*. *aureus* morphology. The bacterial membrane was further destroyed and formed a micelle-like shape compared to the morphology of *S*. *aureus* as shown in Fig. 4 (C), indicating that gA may function like a detergent. Fig. 4 (E) shows that bacteria were completely destroyed and lysed by treatment with gA.

### FT-IR spectra of *S*. *aureus* treated with gA

**Fig 5:**
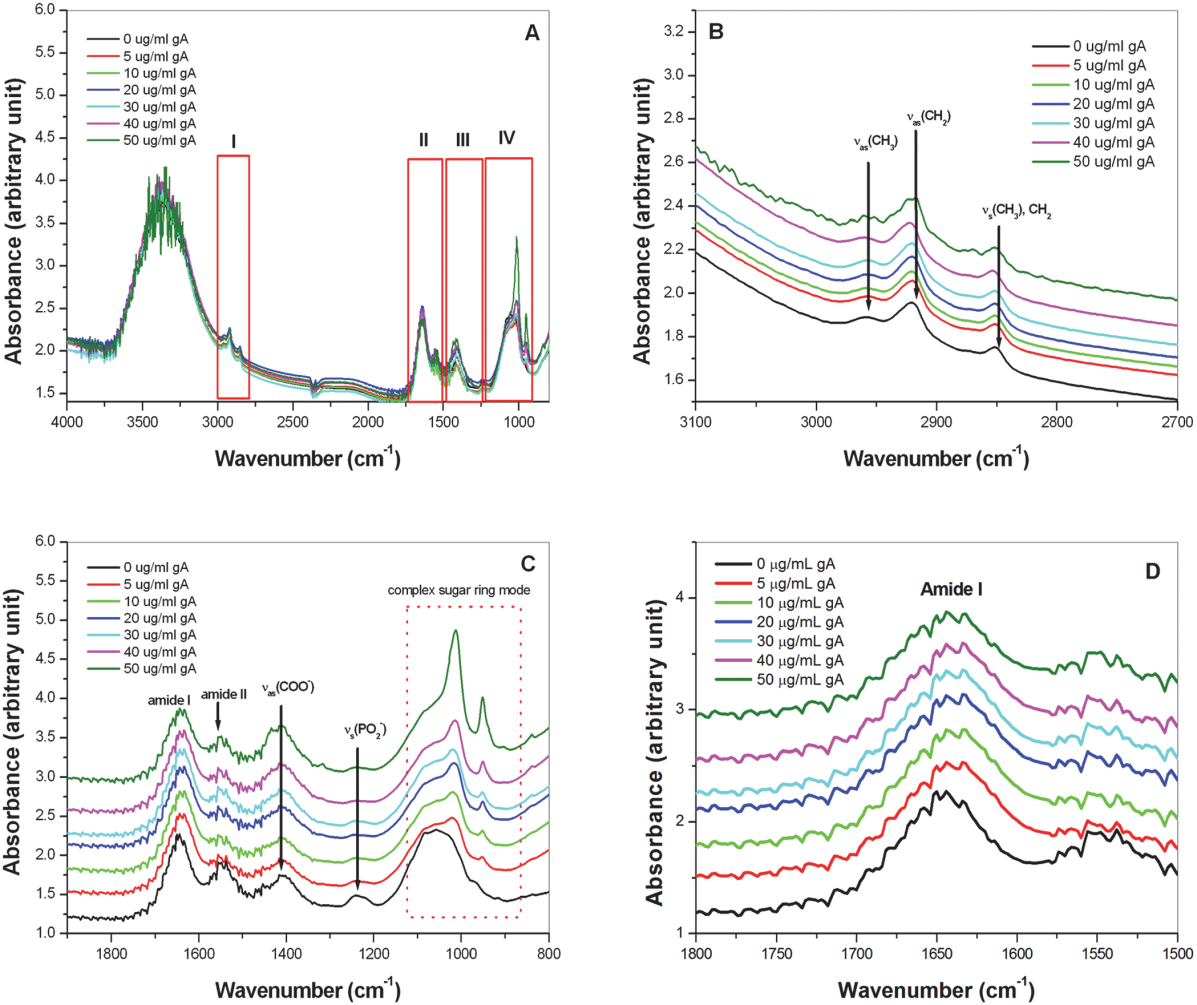
FT-IR spectra of the chemical change in *S. aureus* treated with gA. (A) FT-IR spectra of the chemical change in *S. aureus* treated with different concentrations of gA. In the spectrum, I, II, III, and IV represent the four characteristic IR regions for *S*. *aureus*. (B) FT-IR spectra in region I (C-H vibration of bacterial membrane fatty acids), (C) FT-IR spectra in regions II (protein or peptide amide I and II), III (vibrations of proteins, fatty acids, and phosphate-carrying compounds), and IV (stretching vibration of functional groups of polysaccharides such as C-O), and (D) FT-IR spectra in region II for protein amide I and amide II.

As observed in AFM images, the membrane of *S*. *aureus* was obviously destroyed following treatment with gA. To further understand the chemical composition changes of the destroyed bacterial membrane, FT-IR spectroscopy was used to detect the chemical changes in the *S*. *aureus* membrane. Fig. 5 (A–C) shows the FT-IR spectra of *S*. *aureus* following treatment with different concentrations of gA.

Changes were observed in all four characteristic regions (regions I-IV) of the FT-IR spectrum (Fig. 5 (A)) [36–38]. Region I (3000–2800 cm^−1^) was mainly dominated by the C-H vibration of bacterial membrane fatty acids. In region I (Fig. 5 (B)), three characteristic peaks at 2957 cm^−1^, 2920 cm^−1^, and 2850 cm^−1^ were assigned to the C-H asymmetric (ν_as_(C-H)) or symmetric (ν_s_(C-H)) stretching modes of bacterial membrane fatty acids. Among them, the ν_as_(C-H) band at 2920 cm^−1^ and the ν_s_(C-H) band at 2850cm^−1^ are frequently used to probe the state-of-order of biomembrane [35–37]. The methylene stretching vibrations at 2920 and 2850 cm^−1^ were shifted to a higher wavenumber with an increase in the gA concentration, indicating that the state of *S*. *aureus* membranes transitions from order to disorder.

Region II (1800–1500 cm^−1^) arises from the vibration of amide I and amide II bonds, which are mainly influenced by the amide groups of proteins and peptides. The possible contribution of the amide I and II bands may come from the proteins and peptides of *S*. *aureus* and gramicidin A as well. However, as shown in S2 Fig., the FT-IR spectrum of 1mg/mL gA only in synthetic lipid is different from the FT-IR spectra for *S*. *aureus* treated with 5 μg/mL gA. Therefore, the changes of the amide I band may mainly attribute to the proteins and peptides of *S*. *aureus*. In region II (Fig. 5 (D)), a broad band at 1650 cm^−1^ (amide I) was observed in the absence of gA treatment. This peak shifted to a lower wavenumber (1638 cm^−1^) in the presence of gA. The downshift in the wavelength indicates that the population of proteins underwent a significant alteration, possibly denaturation, with the destruction of *S*. *aureus* [35, 36]. Region III (1500–1200 cm^−1^) was governed by the vibrations of proteins, fatty acids, and phosphate-carrying compounds. In region III (Fig. 5 (C)), two characteristic peaks at 1400 and 1242 cm^−1^ were observed. The former was assigned to the CH_2_ bending vibrations of lipids or proteins, whereas the latter was assigned to the asymmetric stretching mode of the PO^2−^ group of phospholipids or nucleic acids [35–37]. Significant changes were observed at 1242 cm^−1^. The intensity decreased with an increase in the gA concentration, indicating that bacterial membrane phospholipids may be destroyed by treatment with gA.

Region IV (1200–900 cm^−1^) arises from the stretching vibration of functional groups of polysaccharides such as C-O. In region IV, significant changes were observed in the region of 1120–900 cm^−1^ corresponding to (C—O—C) stretching vibration of bacterial membrane glycosidic linkages [36–38]. In the absence of gA, one broad peak centered at 1055 cm^−1^ was observed. With an increase in the gA concentration, this broad peak was gradually separated into a shoulder located at 1090 cm^−1^, a sharp and intense peak located at 1020 cm^−1^, and a small sharp peak located at 950 cm^−1^. The intensity of the 1020 cm^−1^ peak was increased with an increase in the gA concentration. A similar change was also observed for the 950 cm^−1^ peak, and the intensity of this peak was greater with an increase in the gA concentration. The shoulder at 1090 cm^−1^ and the two peaks at 1020 and 950 cm^−1^ were assigned to carbohydrate backbones or complex sugar ring modes [35–37]. The dose-dependent changes in the FT-IR spectra in this region suggested that the bacterial membrane lipid apolar carbon linkages may be destroyed.

## Discussion

The formation of pores and the disintegration of pathogen cell membranes are the most common mechanisms of AMP-induced bacterial killing [3, 9, 14, 18]. Unlike most AMPs, gA is well known to form a single ion channel instead of a pore, which is formed by a bundle of AMPs [27, 28]. Although membrane permeabilization and the dissipation of the electrochemical gradient across the cell membrane are thought to be the underlying mechanisms of gA-induced bacterial killing [27], the bacterial killing mechanism of gA has yet to be studied. In the present study, we examined the antimicrobial activity of gA and studied the effect of gA on the nanostructure and composition changes of *S*. *aureus*.

In general, results show that gA effectively inhibited the growth of *S*. *aureus* at all bacterial growth phases. This is very different from the usual antibiotics, such as β-lactams and quinolones, which require ongoing cell activity and cell division during the bacterial exponential phase for bacteria killing [38, 39]. The minimum effective dose of gA against *S*. *aureus* was approximately 0.5 μg/ml. Although the antimicrobial activity of gA has been associated with membrane permeabilization, the present results further demonstrate that gA can also induce the formation of hydroxyl radicals in a concentration-dependent manner, indicating that gA may perform its bactericidal activity through not only destroying cell membranes, but also by inducing the formation of hydroxyl radicals. Further evidence of hydroxyl radical involving in the bacterial killing is depicted in the S1 Fig. The survival rate of *S*. *aureus* can be rescued by adding hydroxyl radical scavenger, thiourea. This result reinforces that hydroxyl radical induced by gA may play a role on bacterial killing. The effective dose of gA on the induction of hydroxyl radical formation was consistent with the effective dose of gA for killing *S*. *aureus*. Taken together, our results suggest that gA may synergize with antimicrobial activity through hydroxyl radical formation and membrane permeabilization.

A previous study showed that in a comparison of the bacterial killing activity for gD, polymyxin B, hNP-1, and tPMP-1, gD has the lowest permeabilization rate (60–70%) but the highest bacterial killing activity (100%) [30]. The finding that gA can induce the formation of hydroxyl radicals may account for the previous observation. The phenomenon of a low permeabilization rate but high bacterial killing activity induced by gramicidin may be due to the formation of hydroxyl radicals, suggesting that the formation of hydroxyl radicals may play a more important role in the bacterial killing ability of gA than membrane permeabilization.

A possible mechanism of hydroxyl radical production induced by gA may be the disruption of respiration. Previously, several studies have reported that gramicidin disrupts respiration by causing mitochondrial membranes to become permeable to protons [33, 34]. A recent study of the bacterial killing activity of antibiotics has demonstrated that the production of hydroxyl radicals occurs via the Fenton reaction through the reduction of Fe^3+^, the bacterial TCA cycle, and the electron transport chain [19–21]. Our present results clearly demonstrate that the hydroxyl radical formation induced by gA was also associated with disruption of the TCA cycle and a transient depletion of NADH. The O^2−^ radical is produced by the oxidation of NADH via the electron transport chain of the TCA cycle. The induced hydroxyl radicals may further damage DNA, proteins, and lipids, resulting in cell death [40, 41].

The morphology and composition changes of destroyed *S*. *aureus* affected by hydroxyl radicals and membrane permeabilization induced by gA were examined using AFM and FT-IR spectroscopy. From AFM images of *S*. *aureus*, three characteristic morphologies were observed, including pores, a flat shape possibly caused by water permeability, and detergent-like micelles that further led to lysis in the final stage. According to the extent of destruction to the structure of the bacterial membrane, these types of morphology may be representative of the destruction of *S*. *aureus* caused by gA. These destructive stages include the formation of pores at the initial stage, followed by a permeability imbalance that leads to a flattened shape, the formation of a membrane bilayer and resulting detergent-like micelles, and finally lysis.

Among these types of morphology, the observation of pore-like morphology is of particular interest, because gA has been proposed to cause membrane permeation by forming single ion channels, the size of which is too small to be observed with AFM. However, in the present study, the diameter of this pore-like morphology was around 30–94 nm, which far exceeds the size of a single ion channel (4 Å). Therefore, these results suggest that these pores are more likely to be damaged membrane holes, rather than a bundle of aggregated gA peptides. The possible cause of these pores or damaged membranes is most likely hydroxyl radicals. The hydroxyl radicals induced by gA may cause the oxidation of membrane chemical components and induce the formation of pores or damaged membrane holes.

The destructive process of *S*. *aureus* observed with AFM was further characterized using FT-IR spectroscopy. Four features were observed to explain the change in *S*. *aureus* composition during the destruction process: 1) an order-to-disorder transition of membrane fatty acids indicated by a red-shift of the wavelength for methylene stretching vibrations of membrane fatty acids; 2) an order-to-disorder transition of the protein structure indicated by a low-wavenumber shift in amide I; 3) an increase in PO^2−^ intensity possibly arising from either phospholipid head groups or nucleic acids; 4) well resolved peaks for glycoside linkages of polysaccharides in the complex sugar ring mode region. These features indicate that the nanostructure of the cell membrane of *S*. *aureus* underwent an order-to-disorder transition during the bacterial killing process. The chemical composition of the bacterial membrane, including fatty acids, proteins, and carbohydrates, was destroyed as the nanostructure of the bacterial membrane transitioned from order to disorder.

Taken together, the AFM images and the analyses of the chemical composition of the cell membrane with FT-IR spectroscopy suggest that the underlying mechanism of bacterial killing by gA may be due to the formation of hydroxyl radicals, because membrane permeabilization alone may not be sufficient to cause such a full spectrum of bacterial membrane damage. Therefore, although gA induces synergy between hydroxyl radical formation and membrane permeabilization, the hydroxyl radicals may provide the main source of damage to membrane components such as proteins, carbohydrates, and fatty acids, eventually leading to DNA damage [19, 40, 41].

In conclusion, we demonstrated that the antimicrobial activity of gA is associated with not only membrane permeabilization but also the formation of hydroxyl radicals, the latter of which may play a more important role in the bacterial killing activity of gA. The underlying mechanism of hydroxyl radical formation induced by gA may be mediated by disruption of the TCA cycle and a transient depletion of NADH. The effect of hydroxyl radicals on destruction of the bacterial membrane was further studied with AFM and FT-IR spectroscopy. Three stages of gA-induced bacterial membrane destruction were observed with AFM, including pore formation, induction of a permeability imbalance and subsequent formation of a flat shape, and lysis. Destruction of the bacterial membrane involves an order-to-disorder transition of the membrane nanostructure and severe damage to the membrane chemical composition as revealed with FT-IR spectroscopy. The changes in membrane composition include denaturation of proteins, damage to phospholipid polar head groups and fatty acids, and destruction of the glycoside linkages of polysaccharides or nucleic acids.

## Supporting Information

S1 Fig The Growth curves of *S. aureus* following treatment with gA and thiourea. The medium containing *S*. *aureus* in the lag phase was treated without gA (black line), with 150 mM thiourea (green line), 0.1μg/mL gA and 150 mM thiourea (blue line), and 0.1 μg/mL gA (red line). The growth curve of *S*. *aureus* was measured using an Absorbance Reader MRX II (DYNEX) every 30 min at an optical density of 600. Results suggest that the survival rate of *S*. *aureus* treated with gA (induction of hydroxyl radical) was increased by the adding of hydroxyl radical scavenger, thiourea.
(DOCX)

S2 Fig FT-IR spectra for *S. aureus* with or without treatment of gA and gA in synthetic lipids. Compared to the FT-IR spectra of *S*. *aureus* with the treatment of 5 μg/mL gA (red) or without treatment of gA (green), the FT-IR spectrum for gA (1mg/mL) in synthetic lipid, DMPC solution (black) is obviously different. The amide I band for gA in DMPC is located around 1662 cm^-1^, while the amide I band is located at 1638 cm^-1^ and 1650 cm^-1^ for S. aureus treated with 5 μg/mL gA and 0 μg/mL gA, respectively. This indicates that the signal of amide I for *S*. *aureus* contributed from gA may be insignificant. The sample containing gA in DMPC was made by dissolving 1 mg/mL gA with a designed amount of DMPC at a molar ratio of 1:50 in methanol. Then this solution was dried under nitrogen gas. The dried sample was then transferred to 2 mL distilled H_2_O and sonicated for a 10-s pulse each time using the microtip of Branson ultrasonifier at 40°C above the gel-to-liquid crystal transition temperature of DMPC until the solution became transparent (inserting gA in DMPC formed small unilamellar vesicle). This solution was then coated on the ZnSe crystal and dried under nitrogen gas and desiccated under high vacuum for overnight to remove residual solvent. The (ATR)-FT-IR spectrum was measured using a similar protocol as described in the section of material and methods. Curves were smoothed using Savitzky-Golay algorithm.
(DOCX)
