# Supplementary material for: The Antimicrobial Activity of Gramicidin A Is Associated with Hydroxyl Radical Formation
Source: PLoS One. 2015 Jan 26;10(1):e0117065. doi: 10.1371/journal.pone.0117065 (PMC4306519; doi:10.1371/journal.pone.0117065)
Supplement: S1 Fig — The medium containing S. aureus in the lag phase was treated without gA (black line), with 150 mM thiourea (green line), 0.1μg/mL gA and 150 mM thiourea (blue line), and 0.1 μg/mL gA (red line). The growth curve of S. aureus was measured using an Absorbance Reader MRX II (DYNEX) every 30 min at an optical density of 600. Results suggest that the survival rate of S. aureus treated with gA (induction of hydroxyl radical) was increased by the adding of hydroxyl radical scavenger, thiourea. (DOCX) [file pone.0117065.s001.docx]

**Figure S1** **The Growth curves of *S. aureus* following treatment with gA and thiourea.** The medium containing *S. aureus* in the lag phase was treated without gA (black line), with 150 mM thiourea (green line), 0.1μg/mL gA and 150 mM thiourea (blue line), and 0.1 μg/mL gA (red line). The growth curve of *S. aureus* was measured using an Absorbance Reader MRX II (DYNEX) every 30 min at an optical density of 600. Results suggest that the survival rate of *S. aureus* treated with gA (induction of hydroxyl radical) was rescued by the adding of hydroxyl radical scavenger, thiourea.
