# Supplementary material for: The Antimicrobial Activity of Gramicidin A Is Associated with Hydroxyl Radical Formation
Source: PLoS One. 2015 Jan 26;10(1):e0117065. doi: 10.1371/journal.pone.0117065 (PMC4306519; doi:10.1371/journal.pone.0117065)
Supplement: S2 Fig — Compared to the FT-IR spectra of S. aureus with the treatment of 5 μg/mL gA (red) or without treatment of gA (green), the FT-IR spectrum for gA (1mg/mL) in synthetic lipid, DMPC solution (black) is obviously different. The amide I band for gA in DMPC is located around 1662 cm-1, while the amide I band is located at 1638 cm-1 and 1650 cm-1 for S. aureus treated with 5 μg/mL gA and 0 μg/mL gA, respectively. This indicates that the signal of amide I for S. aureus contributed from gA may be insignificant. The sample containing gA in DMPC was made by dissolving 1 mg/mL gA with a designed amount of DMPC at a molar ratio of 1:50 in methanol. Then this solution was dried under nitrogen gas. The dried sample was then transferred to 2 mL distilled H2O and sonicated for a 10-s pulse each time using the microtip of Branson ultrasonifier at 40°C above the gel-to-liquid crystal transition temperature of DMPC until the solution became transparent (inserting gA in DMPC formed small unilamellar vesicle). This solution was then coated on the ZnSe crystal and dried under nitrogen gas and desiccated under high vacuum for overnight to remove residual solvent. The (ATR)-FT-IR spectrum was measured using a similar protocol as described in the section of material and methods. Curves were smoothed using Savitzky-Golay algorithm. (DOCX) [file pone.0117065.s002.docx]

**Figure S2** **FT-IR spectra for *S. aureus* with or without treatment of gA and gA in synthetic lipids.** Compared to the FT-IR spectra of *S. aureus* with the treatment of 5 μg/mL gA (red) or without treatment of gA (green), the FT-IR spectrum for gA (1mg/mL) in synthetic lipid, DMPC solution (black) is obviously different. The amide I band for gA in DMPC is located around 1662 cm^-1^, while the amide I band is located at 1638 cm^-1^ and 1650 cm^-1^ for S. aureus treated with 5 μg/mL gA and 0 g/mL gA, respectively. This indicates that the signal of amide I for *S. aureus* contributed from gA may be insignificant. The sample containing gA in DMPC was made by dissolving 1 mg/mL gA with a designed amount of DMPC at a molar ratio of 1:50 in methanol. Then this solution was dried under nitrogen gas. The dried sample was then transferred to 2 mL distilled H_2_O and sonicated for a 10-s pulse each time using the microtip of Branson ultrasonifier at 40°C above the gel-to-liquid crystal transition temperature of DMPC until the solution became transparent (inserting gA in DMPC formed small unilamellar vesicle). This solution was then coated on the ZnSe crystal and dried under nitrogen gas and desiccated under high vacuum for overnight to remove residual solvent. The (ATR)-FT-IR spectrum was measured using a similar protocol as described in the section of material and methods. Curves were smoothed using Savitzky-Golay algorithm.
